# Building Self-Efficacy in Parenting Adult Children With Autistic Spectrum Disorder: An Initial Investigation of a Two-Pronged Approach in Role Competence

**DOI:** 10.3389/fpsyg.2022.841264

**Published:** 2022-07-22

**Authors:** Cecilia Nga Wing Leung, Brenda Tsang, Doris Haiqi Huang, Raymond Won Shing Chan

**Affiliations:** New Life Psychiatric Rehabilitation Association, Hong Kong, Hong Kong SAR, China

**Keywords:** autistic spectrum disorder (ASD), parenting self-efficacy, mindfulness, adult, Chinese, parent

## Abstract

Previous studies on parenting adult children with ASD were scarce, and their intervention protocols mainly were derived from established work with children. Development of an applicable adult-oriented protocol and demonstration of its effectiveness is warranted. The present study outlined the development and evaluation of Core Autism Parenting Skills (CAPS), which targets to enhance parenting self-efficacy (PSE) intervention for adult children with ASD by addressing two intervention goals in parallel: acquisition of parenting skills and cultivating positive attributes. In CAPS, PSE is operationalised into four parent roles: to observe, reinforce, empathise, and accompany, each with requisite attributes, skills, and prescribed training. Twenty-seven parents with adult children with ASD (aged 16–37) were recruited. They completed measures assessing their PSE, competence in the four parent roles, and emotional well-being at pre-training, post-training and 2-month follow-up. The intervention was well-received by the participants and reported significant improvements in PSE, parent role competence at post-training and 2-month follow-up. The applicability of PSE and parent role competence in constructing effective parenting intervention for adult children with ASD was supported.

## Introduction

Ample studies suggested that parents of children with Autism Spectrum Disorder (ASD) are susceptible to elevated parenting stress, even compared with children with other developmental disabilities (Hayes and Watson, 2013). They tend to report elevated rates of anxiety and depression, poorer quality of life and overall psychological well-being (Weitlauf et al., 2014; Pisula and Porêbowicz-Dörsmann, 2017). Impact on parents’ personal life also extends to occupational, social and recreational areas (Smith et al., 2008; Nealy et al., 2012).

ASD-specific symptom severity (i.e., impairments in social communication and interaction and restricted patterns of behaviours) and co-occurring behavioural problems are suggested to be the two most decisive factors in contributing parental distress and negative parenting behaviours (Dieleman et al., 2018). ASD symptoms and behaviour problems in adolescents and adults with ASD tend to persist or shift in presentation, continuously influencing the well-being of mothers (Orsmond et al., 2006). Even with maturity in age, grown children with ASD continually rely on parents for medical, academic and daily living arrangements (Kanne et al., 2011; Hume et al., 2014; Strunk et al., 2014). A 10-year follow-up study showed that maternal anxiety about the child’s day-to-day activities and care arrangements subsides when a grown child moves out. However, maternal depressive symptoms remain stable in those who had previous concerns about their child’s transition to adult independence (Barker et al., 2011). Parents continue to be the primary source of support and care for their adult children, having high childcare responsibility and involvement and experiencing chronic parenting stress throughout mid-age to advanced age (Barker et al., 2011).

Moreover, the developmental need for greater autonomy in grown children may easily conflict with the parent’s guidance. A diary study revealed that parents of adolescents with ASD experience more frequent arguments, on top of other daily challenges such as increased time for caretaking and intrusions of work time (Smith et al., 2010). In adulthood, mother-child interactions are often more aversive, resulting in negative family dynamics and recurrent conflicts (Golan et al., 2018). Maternal well-being and mother-child interaction created a vicious cycle: maternal pessimism about the child’s future maintained the harsh feelings between parents and child or within the parents themselves; negative mother-child interaction prevents effective parenting to be implemented, maintaining their hopelessness (Orsmond et al., 2006).

In order to empower parents’ coping to the life-long parenting stress, research attention was drawn to psychosocial factors that support parenting resilience (Hayes and Watson, 2013; Prata et al., 2019). Parenting self-efficacy (PSE), described as a “parent’s belief in his or her ability to influence the child and his or her environment to foster the child’s development and success” (Ardelt and Eccles, 2001, p. 945), is a psychosocial factor that is associated with adaptive parenting, and well-being in parents and children (Albanese et al., 2019). In mothers with children with ASD, higher PSE in parents is associated with decreased parental anxiety, depression, and child behavioural problems (Hastings and Brown, 2002; Kuhn and Carter, 2006; Weiss et al., 2012). Research also suggested that PSE can be enhanced through professionally supported intervention, translating into improved child, parent and service outcomes (Keen et al., 2010).

In the field of ASD, recent discussions on intervention for PSE advocated a two-pronged approach comprised of (1) problem-focused strategies in remediating problem behaviours and (2) strategies to promote psychological acceptance for situations beyond control (Weiss et al., 2016). The rationale of the first prong of intervention could be attributed to the role of the environment and people around individuals with ASD in maintaining behavioural problems and evoking negative responses (Horner et al., 2002). Being the people they most often are rewarded by, parents and the family members serve as one of the most substantial reinforcing factors to problem behaviours (Reeve and Carr, 2000). Equipping parents with problem-focused strategies could improve the generalizability of skills, intensify treatment and augment treatment outcomes (Anderson and Romanczyk, 1999). Nevertheless, building self-efficacy in parents requires skill acquisition and, more importantly, nurturing acceptance, hopefulness, compassion and endurance, which is advocated in the second prong of intervention. Individuals with ASD can be challenging to understand due to their atypical social responsiveness and maladaptive behaviours (Falk et al., 2014). When being confronted with these challenges, parents may not receive clear reinforcement in terms of “positive” changes in their child’s behaviour or demeanour. They may start to perceive themselves as ineffective parents. Parents’ capacity in adhering to behavioural treatment programmes could be further reduced by their mental health problems (Moore, 2009) and loss of hope after observing regression in some critical skills in their children (Bashir et al., 2014). Under these circumstances, providing parents with new behavioural management plans may not affect them if they already perceive themselves as ineffectual (Falk et al., 2014). Cultivating positive attributes and psychological acceptance in the face of setbacks and regressions are thus necessary for promoting PSE among parents of children with ASD.

Notably, existing intervention for parents with adolescents and adults with ASD was not developed to enhance PSE. For instance, some targeted at enhancing children’s social skills (Schultz et al., 2012), parents’ overall emotional well-being (Singh et al., 2014) or family communication (Golan et al., 2018). Intervention targeting PSE are scarce. The research team aimed at supplementing the above intervention gaps by developing a training intervention focusing on PSE for parents with adult ASD children. We started by operationalising PSE as the acquisition of competence of four parent roles derived from our clinical experiences and about the role theory, positive behavioural support, mindfulness, developmental psychology, and family life cycle. The four critical parent roles are summarised below:

### To Observe

As discussed above, parents’ sense of hope in soliciting children’s change is fundamental to effective behavioural treatment. Loss of hope is particularly prominent as children reach adulthood. Maturation in age does not bring hope in increased independence, but saturation in growth and need for autonomy; both are obstacles to adhering to parental instructions and changing one’s behavioural pattern, creating parent-child conflicts. As negative interaction accumulates, parents commonly present with a fixated, negative perception toward their adult children, such as “they always bring inconvenience,” “they can never change,” maintaining negative interaction and feeling of hopelessness. Fundamental to any skill acquisition, parents need to nurture an open, curious and non-judgemental attitude in observing their children in order for them to interrupt the vicious cycle of negative interactions and hopeless feelings. These positive attributes echo the concept of mindfulness, which involves a non-judgmental observation of the ongoing stream of internal and external stimuli in the present moment and adopting an attitude of curiosity, openness, and acceptance toward one’s own experience (Bishop et al., 2004).

### To Reinforce

Reinforcement learning strategies are widely used in ASD intervention and have demonstrated robust effectiveness across different age ranges (Schuetze et al., 2017). Parents need to equip with appropriate behavioural management skills on the adolescents’ disruptive behaviours and reinforce adaptive ones (Burrell and Borrego<suffix>Jr.</suffix>, 2012). To parents, challenges in delivering reinforcement move beyond the technical aspects: they tend to present with a biased perception overemphasising inadequacies, regression and negativity toward their children, contributing to a frequent complaint that there is no opportunity to give reinforcement. This phenomenon may be particularly prominent in Asian communities due to cultural influences and the frequent use of authoritarian parenting styles (Shen et al., 2018). In order to equip parents to be an effective interventionist, parent training should also harbour positivity and address the biased perception toward their adult children before any reinforcement techniques could be implemented.

### To Empathise

Individuals with ASD commonly present with a high co-occurrence of mental health problems in adulthood (Volkmar et al., 2017). Alexithymia, defined as a deficit in identifying and describing one’s own emotions and feelings (Taylor, 1984), has been recognised as a contributing factor of mental health problems in individuals with ASD (Gaigg et al., 2018; Morie et al., 2019). Individuals with alexithymia need others to name their emotions and facilitate appraisals toward triggering events (Vanheule et al., 2011). While regulating emotions in individuals with ASD is assumed mainly by parents, parents themselves also exhibited alexithymia traits (Szatmari et al., 2008; Durukan et al., 2018). Inability to understand and express emotional states in parents leads to a reduced capacity to contain the children’s emotions, reduce parental reflective functioning, and hinder positive relationships with their children (Yürümez et al., 2014; Ahrnberg et al., 2021). Parents are therefore in need of training to enhance their emotional sensitivity toward self and others and techniques in delivering empathic responses to the children to co-regulate their negative emotions in times of stressful events.

### To Accompany

The relationship between parents and children does not only impact on the well-being of both but also the effectiveness of any parent-mediated intervention. ASD was found to be associated with the increased likelihood of a disordered parent-children relationship characterised by high expressed emotions (Smith et al., 2010). Relatedly, family leisure activities are often sacrificed for various reasons related to the diagnosis (Nealy et al., 2012). Both reduce the joy that family members can experience in the family context. Repairing the communication pattern and fostering positive emotional exchange in the parent-child relationship would facilitate the functioning of the individual, the parent and the family to navigate various stressors in the life journey. Furthermore, the developmental nature of ASD poses tremendous demand in the resilience of parents, which depends on positive relationships with themselves and external resources (Bronfenbrenner and Ceci, 1994). Self-compassion, defined as self-kindness, common humanity and mindfulness (Neff, 2003), emerges as a powerful source of resilience in the face of stressors. Self-compassion often leads to greater perceived confidence, less fear of failure and more persistence and goal reengagement after failure (Neff et al., 2005; Breines and Chen, 2012), which are crucial attributes in accompanying a child with ASD in his/her growth, and should be cultivated in intervention for parents of children with ASD (Bohadana et al., 2019).

The current paper outlined the development of parent training, the Core Autism Parenting Skills (CAPS), which aims at enhancing the PSE of parents of adults with ASD by improving the competence of the four parent roles. It was hypothesised that the CAPS would demonstrate (1) acceptability as measured by group attendance and homework compliance; (2) effectiveness in enhancing PSE of parents and the attributes and skills in the four parent roles, and (3) spill-over effectiveness in reducing negative mood and parenting stress.

## Materials and Methods

### Participants

The parents eligible for this study shall fulfil these inclusion criteria: (a) have a child aged 15 or above, with a confirmed ASD-related diagnosis in DSM-IV or DSM-5 by a registered psychiatrist and clinical psychologist, including Autistic Disorder, Pervasive Developmental Disorder-Not Otherwise Specified (PDD-NOS), Asperger’s Syndrome, Autism Spectrum Disorder or Social Communication Disorder; and with an FSIQ of 70 or above, as measured by local standardised intelligence tests, i.e., the Wechsler Intelligence Scale for Children-Fourth Edition (Hong Kong) or the Wechsler Adult Intelligence Scale-Fourth Edition (Hong Kong); (b) living with the children at the time of intervention; (c) ethic Chinese and are native speakers of Cantonese, a local dialect of Southern China. The chosen sample is a convenient sample from a community centre primarily serving individuals with ASD and an FSIQ of 70 or above.

**FIGURE 1:**
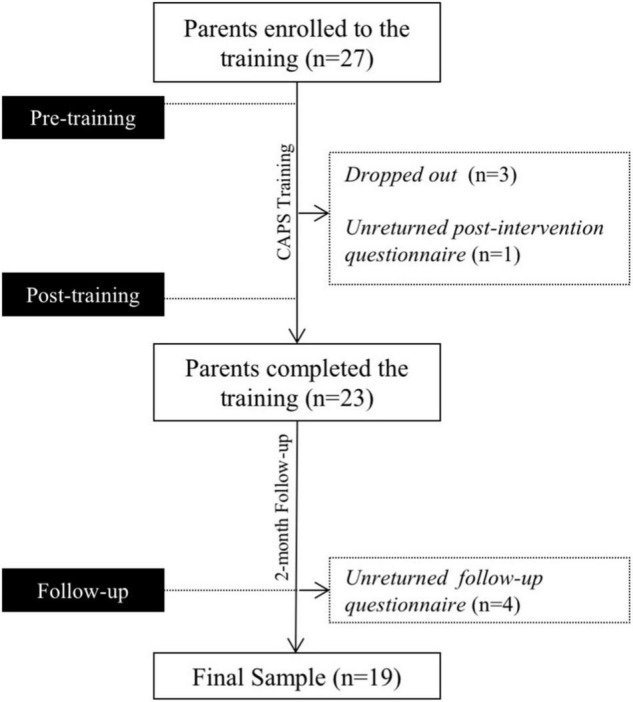
Participant flow of the study.

Twenty-seven eligible parents enrolled in the study. Three dropped out due to change in engagements and family circumstances. The participant flow of the study is illustrated in Figure 1. All missing data were excluded from the study. Twenty-three parents (6 fathers and 17 mothers) completed the intervention. All of the parents are biological parents of their children. They are parents of 21 persons with ASD (PWAs). The average age of the PWAs (*n* = 18 males) was 24.33 years (SD = 5.89, range 16–37). Demographic information about parents and the PWAs is reported in Table 1.

**TABLE 1:** Demographic information of parents and persons with ASD (PWAs).

Parent characteristics	*n* = 23
Sex	
Mother (*n* = 17)	73.9%
Father (*n* = 6)	26.1%
Age	
45–49	21.7%
50–54	26.1%
55–59	17.4%
60–64	21.7%
65–69	8.7%
70 or above	4.3%
Education level	
Primary	4.3%
Junior secondary	52.2%
Senior secondary	13.0%
Tertiary or above	30.4%
Employment status	
Full-time employment	47.8%
Part-time employment	13.0%
Home-maker	17.4%
Retired	21.7%

**PWA characteristics**	***n* = 21**

Sex	
Female (*n* = 3)	14.3%
Male (*n* = 18)	85.7%
Age (years)	
Mean (SD)	24.33 (5.89)
Range	16–37
Family composition	
Two parents	95.2%
Single parent	4.8%
ASD diagnosis	
Autistic Disorder/Autism	42.9%
Asperger’s Syndrome	14.3%
Autism Spectrum Disorder	42.9%
IQ range	
Borderline	38.1%
Low average	19.0%
Average	33.3%
High average	9.5%
Psychiatric comorbidity	
ADHD	28.6%
Anxiety	23.8%
Depression	4.8%
Psychosis	14.3%

### Procedures

#### Study Approval

The study had been reviewed and received ethical approval with reference to the Declaration of Helsinki (2013) from the hosting organisation. Informed consent to the study was obtained from the parents.

#### Intervention Development

The procedures for developing CAPS referenced the cultural adaptation model (Domenech Rodriìguez and Weiling, 2004), which included balancing community and scientific needs, adapting appropriate evaluation measures and developing a culturally sensitive packaged intervention. The development of the CAPS was guided by scientific, community and professional inputs. The research team consisted of a panel of clinicians, including clinical psychologists, counselling psychologist and social workers, who have rich experience working with families with adolescents and adults with ASD. The protocol has been revised after collecting quantitative and qualitative feedback from families who participated in previous versions. The steps for protocol development were as follows.

##### Consolidating Theoretical Basis

A systematic search and review of existing literature and treatment protocols were conducted to examine the conceptual framework, programme designs and effectiveness of existing programmes delivered to parents of children with ASD. Parent interventions were often delivered in conjunction with social competence training (Lopata et al., 2010; Thomeer et al., 2012) or cognitive behavioural therapy (Storch et al., 2013; Wood et al., 2014) to enhance children’s outcomes. Some intervention focusing on parental outcomes included PSE as an auxiliary outcome (e.g., Iadarola et al., 2018; Kuravackel et al., 2018), but their development was not oriented to enhance PSE. The concept of PSE emerged from social cognitive theory. PSE represents a domain-specific subset of self-efficacy and is found to be associated with adaptive parenting, psychosocial adjustment, and well-being in parents and children (Albanese et al., 2019). In the ASD population, higher PSE in parents is associated with lower negative mood, more effective coping strategies, higher agency in the parenting role and better family resilience (Kurzrok et al., 2021). Kurzrok et al. (2021) also suggested that as self-efficacy is highly domain-specific, its assessment should be tailored to the specificity. Kurzrok et al. (2021) and colleagues developed a measurement on autism-specific parenting self-efficacy (PSEa) in response to the specific need of the ASD population.

##### Defining and Operationalizing the Four Parent Roles

Critical parent roles that are fundamental to PSEa were discussed among the research team by referencing our own clinical experiences and existing literature. The four roles: to observe, reinforce, empathise, and accompany were chosen with a balanced consideration of the developmental needs of adolescents and adults with ASD and skills delivered in the existing parent-mediated intervention of ASD. Competence of each role was then operationalized by first, the fundamental attributes necessary to each role (e.g., positivity shall be fundamental to the role of “to reinforce”), and then, the skills crucial to the role (e.g., giving praises was crucial to the role of “to reinforce”). The four roles were then sequenced in consideration of scaffolding relevant parenting skills.

##### Designing Training Method and Session Content

An eight-session group intervention was proposed for the acquisition of the competence in four parent roles. Training on each role comprises two sessions, with the first session focusing on cultivating positive attributes and the second on acquiring parenting skills. Further, to cultivate self-compassion throughout the training, guided reflection was designed in weekly home practices to guide parents applying positive attributes of the four parent roles, including non-judgement, positivity, validating own needs and seeking support, on themselves. Lastly, in-session mindfulness practices echoing each session theme were tailored with reference to mindfulness interventions in Dialectical Behaviour Therapy (Linehan, 2015) and Mindful Parenting protocols (Bögels and Restifo, 2013). Home practices for each session were then designed with a principle to consolidate the attributes/skills delivered and the mindfulness practice. Training methods in the CAPS largely followed a combination of didactic teaching, discussions, guided practices and home assignments. Ample clinical case scenarios and demonstration videos were used throughout the course to facilitate learning.

##### Manualising the Training

The CAPS was manualised and trial-run to test its feasibility. Members of the research team conducted pilot groups to test out the feasibility of protocol and strengthen staff competence. Feedback from parents was collected for further review and modifications. For instance, parents were observed to have difficulty picturing scenarios in illustrations and role-play practices. In response, all scenarios designed in the protocol were video-filmed.

To ensure fidelity and facilitate review discussion among the team, all pilot and data collection sessions were video-recorded for supervision and peer review among the research team.

##### Validating With Local Assessment Tools

The Autism-Specific Parenting Self-Efficacy Scale (PSEaS; Kurzrok et al., 2021) was employed as the primary measure considering its theoretical origin emphasising PSE in parents of children with ASD. For local use, the PSEaS was translated and back-translated for standardisation in Chinese before application. A self-developed questionnaire was employed to assess the attributes and skills of the four operationalised parent roles. Three subscales in the Multidimensional Assessment of Parenting Scale (MAPS; Parent and Forehand, 2017) were selected to supplement the assessment of changes in parenting. Locally-validated questionnaires were chosen to assess the mood functioning and parenting stress of the parents.

#### Intervention Setting

All interventions were carried out in a community-based centre serving PWAs aged 15 or above in Hong Kong. PWAs were either self-referred or referred by local psychiatric centres or mental health service units for services. All PWAs applied for services, and their parents were interviewed jointly by their case manager at intake. All parents were introduced to the CAPS at intake or subsequent phone interviews if the parents were not available to join the intake. Parents interested in the CAPS were put on a waitlist and contacted to confirm enrolment before groups started. Each intervention group comprised 7–10 parents. The programme consisted of 8 weekly sessions; each lasted 2 h. The session outline of the CAPS is summarised in Table 2. The pre-intervention assessment was mailed to parents 1 week prior to the first session and was collected at the first session. The post-intervention assessment was collected at the last session of the intervention. The follow-up assessment was collected at a reunion session 2 months after the last session or by mail for absentees of the reunion session. All intervention was conducted in 2021 and delivered in-person.

**TABLE 2:** Session outline of the CAPS.

Session	Module	Attributes	Skills	Mindfulness practices
1	To observe	Curiosity Patience Non-judgemental	N/A	Mindful breathing- Counting
2		N/A	To practice deep breathing To be aware of hot cognitions during triggering incidents To reflect on the biased assumption on the PWA To respond to PWA’s behaviours in a non-reactive way	Mindful seeing – Observing a photo of my child
3	To reinforce	Positivity Reasonable expectation	N/A	Mindful positivity – Sending encouragement to my child
4		N/A	To describe behaviour in observable terms To choose suitable reinforcement, including praises and preferred activities To reinforce timely	Mindful positivity – Revisiting a positive exchange with my child
5	To empathise	Emotional awareness Emotional containment	N/A	Mindful emotion – Experiential practice of accepting negative emotions in parenthood
6		N/A	To name the emotions of PWA To understand the psychological needs of PWA To guide PWA in problem-solving	Mindful emotion – Experiential practice of accepting negative emotions in parenthood
7	To accompany	Compassion Hopefulness	To understand the non-linearity of growth in PWA To develop habits of chatting and family time to strengthen positive emotional exchange	Mindful compassion – Loving Kindness Meditation to parent and child
8	Integration	N/A	To review learning and skills taught in the group To consolidate parents’ changes and progress	Mindful compassion – Building positive resources in life journeying

### Measures

#### Attendance and Homework Compliance

The attendance rate of each parent was calculated by having the percentage of the total number of sessions attended to the eight sessions in total. Homework was assigned in the first seven sessions. Homework compliance was reflected in the homework submission rate of each parent, calculated by having the percentage of the total number of homework submitted to the seven homework in total.

#### Primary Measures

##### Autism-Specific Parenting Self-Efficacy Scale

The PSEaS assesses parents’ confidence in parenting a child with ASD. It consists of 17 items on a 5-point Likert scale from 1 to 5, with a higher score suggesting higher confidence (Kurzrok et al., 2021). The scale demonstrated good internal consistency (Cronbach’s alpha = 0.91). An example item of PSEaS would be “I feel confident that I can handle difficult moments with my child (for example: support my child when the predictable routine changes, make things fun even when they are unexpected, navigate major transitions in interventions or school).” Translation of PSEaS to Chinese was done by a team of trainee clinical psychologists, following the standard translation and back-translation procedure. The Chinese version was finalised using a panel consensus approach after reviewing discrepancies between the original, the translation and the back-translation. The current study used the average score of all 17 items as the outcome variable for analysis.

##### A Self-Developed Questionnaire on Attributes and Skills of the Four Parent Roles

The attributes and skills of the four parent roles (ASPR) was developed in reference to the attributes and skills operationalised in the training. It consists of 17 items rated on a 10-point Likert scale from 1 to 10, with higher scores indicating higher competence. The 17 items are divided into five domains, with the first four on the four parent roles, respectively. The last domain assesses whether parents apply the attributes cultivated in the four parent roles, including non-judgemental, positivity, validating own needs and seeking support on themselves. Examples of questionnaire items in respective domains included “I can observe my child in a curious attitude; I am interested in knowing his/her actions and thoughts”; “I can choose and apply reinforcement effectively when my child behaves appropriately”; “I can name and reflect my child”s feelings when they are displaying emotions, for example, “I noticed that you are angry right now”; “I notice my child’s progress even if it is small. I understand that improvements have to be accumulated, and there is regression at times” and “I recognise my contribution to the family”. Average scores of all questions in each domain were used as the outcome measures.

##### The Multidimensional Assessment of Parenting Scale

The MAPS is a 34-item questionnaire assessing parenting practices with strong psychometric properties reported in both the original and Chinese versions (Parent and Forehand, 2017; Ahemaitijiang et al., 2021). Given the relevancy to the CAPS and applicability to the developmental stage of the sample, three subscales, namely, “Proactive Parenting,” “Positive Reinforcement” and “Supportiveness,” were chosen to be outcome measures of the current study. Each question was rated on a 5-point Likert scale from 1 to 5, with a higher score suggesting more frequent use of measured parenting practices.

#### Secondary Measures

##### Depression Anxiety and Stress Scales-21

The Depression Anxiety and Stress Scales-21 (DASS-21) is a validated tool on assessing depression, anxiety, and stress with high internal consistency and construct validity (Henry and Crawford, 2005). The Chinese Version of DASS-21 adopted a Likert rating scale from 0 to 3, with higher scores indicating higher symptom severity (Taouk et al., 2001). The average ratings of each of the three syndromes were used as the outcome variables.

##### Parental Stress Scale-Chinese Version

The PSS assesses parental stress with 17 items on a 6-point Likert scale, with a higher score suggesting higher parental stress. Its original and Chinese versions demonstrated good reliability (Berry and Jones, 1995; Cheung, 2000). In the current study, the average score of all items was used as the outcome variable.

### Data Processing

Data of withdrawn participants was removed from analysis following the per-protocol principle. All statistical analysis in this study was conducted using IBM SPSS Statistic 24.

## Results

### Attendance and Homework Compliance

The mean attendance rate of all parents included in the analysis was 85.33% (SD = 13.41%). The mean homework submission rate of all parents included in the analysis was 60.25% (SD = 37.54%). The frequency distribution of the two variables is summarised in Table 3 below.

**TABLE 3:** Frequency distribution of attendance rate and homework submission rate.

	*n* = 23
*Attendance rate (Mean = 85.33%; SD = 13.41%)*	
0–50%	4.3%
51–75%	30.4%
76–100%	65.2%
*Homework submission rate (Mean = 60.25%; SD = 37.54%)*	
0–50%	30.4%
51–75%	26.1%
76–100%	43.5%

### Primary Measures

Repeated-measure ANOVA was conducted to assess intervention effect across three time-points, pre-intervention (T1), post-intervention (T2), and 2-month follow-up (T3) in all questionnaire measures. *Post hoc* analyses with a Bonferroni adjustment from T1 to T2 and from T1 to T3 were conducted. Descriptive statistics and effect sizes of all measures were summarised in Table 4. On PSEaS, significant difference across three time-points was found [*F*(2,28) = 5.69, *p* = 0.011] with a significant improvement from T1 (*M* = 3.51, SD = 0.39) to T2 (*M* = 3.93, SD = 0.44; *p* = 0.012) indicated in *post hoc* analysis. For the role “to observe” in ASPR, significant difference across three time-points was reported [*F*(2,36) = 5.87, *p* = 0.007]. *Post hoc* analysis indicated significant improvement from T1 (*M* = 4.74, SD = 1.68) to T2 (*M* = 6.24, SD = 1.76; *p* = 0.015). For the role “to reinforce” in ASPR, significant difference across three time-points was reported [*F*(2,36) = 6.32, *p* = 0.007]. *Post hoc* analysis indicated significant improvement from T1 (*M* = 5.56, SD = 1.61) to T2 (*M* = 6.65, SD = 1.50; *p* = 0.029) and from T1 to T3 (*M* = 6.46, SD = 1.71; *p* = 0.038). For the role “to empathise” in ASPR, significant difference across three time-points was reported [*F*(2,36) = 5.24, *p* = 0.020]. *Post hoc* analysis indicated significant improvement from T1 (*M* = 5.08, SD = 1.86) to T3 (*M* = 6.18, SD = 1.46; *p* = 0.010). For the role “to accompany” in ASPR, repeated-measure ANOVA did not identify significant difference across three time-points [*F*(2,36) = 1.20, *p* = 0.305]. For the self-application domain in ASPR, significant difference across three time-points was reported [*F*(2,36) = 5.85, *p* = 0.013]. *Post hoc* analysis indicated significant improvement from T1 (*M* = 4.87, SD = 1.99) to T3(*M* = 6.47, SD = 1.66; *p* = 0.008). On MAPS, significant difference across three time-points was found in the Positive Reinforcement subscale [*F*(2,36) = 14.68, *p* < 0.001], with significant improvements from T1 (*M* = 3.27, SD = 0.67) to T2 (*M* = 3.75, SD = 0.56; *p* = 0.004) and from T1 to T3 (*M* = 3.88, SD = 0.62; *p* = 0.001) indicated in the *post hoc* analyses. There were no significant difference in the Proactive Parenting subscale [*F*(2,36) = 0.67, *p* = 0.498] and Supportiveness subscale [*F*(2,36) = 1.77, *p* = 0.188] across three time-points.

**TABLE 4:** Means and changes in outcome variables across timepoints.

	Mean (SD)			Partial Eta Sq	Cohen’s *d*	
Variables	T1 (*n* = 23)	T2 (*n* = 23)	T3 (*n* = 19)	T1–T2–T3	T1–T2	T1–T3
PSEaS	3.51 (0.39)	3.93 (0.44)	3.70 (0.37)	0.29*	1.01*	0.50
ASPR – To observe	4.74 (1.68)	6.24 (1.76)	5.96 (1.99)	0.25**	0.87*	0.66
ASPR – To reinforce	5.56 (1.61)	6.65 (1.50)	6.46 (1.71)	0.26**	0.70*	0.54*
ASPR – To Empathise	5.08 (1.86)	6.24 (1.77)	6.18 (1.46)	0.23*	0.64	0.66**
ASPR – To Accompany	5.87 (1.90)	6.50 (1.85)	6.54 (1.63)	0.06	0.34	0.38
ASPR – Self application	4.87 (1.99)	6.14 (1.72)	6.47 (1.66)	0.25*	0.68	0.87**
MAPS proactive parenting	3.56 (0.55)	3.58 (0.66)	3.66 (0.49)	0.04	0.03	0.19
MAPS positive reinforcement	3.27 (0.67)	3.75 (0.56)	3.88 (0.62)	0.45***	0.23**	0.95***
MAPS supportiveness	3.29 (0.54)	3.58 (0.66)	3.60 (0.60)	0.09	0.48	0.54
DASS depression	0.59 (0.61)	0.45 (0.48)	0.39 (0.37)	0.09	0.26	0.40
DASS anxiety	0.55 (0.63)	0.41 (0.45)	0.35 (0.35)	0.02	0.26	0.39
DASS stress	1.01 (0.72)	0.90 (0.65)	0.82 (0.58)	0.01	0.16	0.29
PSS-C	3.36 (0.72)	3.17 (0.58)	2.97 (0.53)	0.11	0.31	0.62

*p ≤ 0.05; **p ≤ 0.01; ***p ≤ 0.001. PSEaS, Autism-Specific Parenting Self-Efficacy Scale; ASPR, Self-developed questionnaire on attributes and skills of the four parent roles; MAPS, Multidimensional Assessment of Parenting Scale; DASS-21, Depression Anxiety Stress Scale-21; PSS-C, Parental Stress Scale-Chinese Version.

### Secondary Measures

There were no significant difference across three time-points in the any of the subscales of DASS-21, [Depression: *F*(2,36) = 1.71, *p* = 0.200; Anxiety: *F*(2,36) = 0.27, *p* = 0.680; Stress: *F*(2,36) = 0.19, *p* = 0.811], or PSS-C [*F*(2,36) = 2.29, *p* = 0.121].

## Discussion

The current study examined the acceptability and effectiveness of the Core Autism Parenting Skills (CAPS) in enhancing parenting self-efficacy (PSE) of parents with adult ASD children by improving the core competence of four parent roles: to observe, reinforce, empathise and accompany. The acceptability of the intervention was reflected in the mean attendance rate as high as 85%. The high attendance rate reflected that parents were motivated and receptive toward the CAPS training. Despite achieving a lower mean rate than attendance, the homework submission mean rate was skewed toward the higher end: about 70% of the parents submitted at least half of the assigned homework. Satisfactory homework compliance reflected parents’ eagerness in practising the skills in daily lives despite heavy caretaking responsibility and daily hassles.

On the effectiveness of the CAPS, significant post-intervention improvement was reported in PSE and the roles to observe, reinforce, and empathise measured by the ASPR. A relatively high score in the role “to accompany” on ASPR at pre-intervention (5.87) may have made statistically significant change more difficult even the scores rose numerically to 6.50 and 6.54 at post-intervention and follow-up, respectively. Significant changes in the first three roles at post-intervention suggested that training delivered in CAPS effectively enhanced role competence. Specifically, the training method in CAPS first focused on cultivating positive attributes then equipping parenting skills. The demonstrated gains in the CAPS supported the two-pronged intervention approach for parent intervention in ASD, as discussed in Weiss et al. (2016), promoting psychological attributes and acquiring problem-focused strategies. Applying the two-pronged intervention in CAPS may also echo the emerging trend of integrating treatment originated from behavioural principles and mindfulness-based intervention to complement each other. For instance, a mindfulness-based positive behavioural support program*me* was developed to address the limitation that parents learning only behavioural approaches may quit easily due to decreasing motivation and implementation fatigue (Singh et al., 2019).

Another promising finding was that parents reported being abler to cultivate positive attributes toward themselves, potentially reflecting better self-awareness and self-compassion. Parents with children with disabilities often put the needs of their children, instead of their own, at a higher priority, which commonly leads to burnout and exhaustion (Nealy et al., 2012). Paradoxically, parents sacrificing their well-being for caretaking tasks did not promote children’s well-being but was detrimental to their resilience and overall family functioning (Pisula and Porêbowicz-Dörsmann, 2017). The CAPS attempted to address the lack of self-care commonly observed in parents of individuals with ASD by relating the positive attributes in the four parent roles on themselves. A change in relationship with self may potentially enhance their resilience and self-compassion, facilitating their acquisition and continued practices of the attributes and skills shared in the CAPS training.

The results on spill-over effectiveness in reducing negative mood and parenting stress in parents were contrary to our hypothesis. All variables did not demonstrate significant improvement at post-intervention. Notably, the divergence in outcomes of parents’ emotional well-being and PSE in the current study may support separate psychological constructs for intervention. Reduction in parenting stress was commonly reported in other parent interventions, especially for parents with younger children (see review Lichtle et al., 2020). On the contrary, parents with adult children may have suffered from stress more chronically and are less likely to show a significant decrease of negative mood within 8 weeks. In our previous studies on social competence training for individuals with ASD, parents of adolescent participants showed a significant reduction in parenting stress but not parents with adult children (Chan et al., 2018; Leung et al., 2019). Possibly, for parents with adult children to alleviate their negative mood, they require more intensive and long-term intervention.

The CAPS enriched the existing parent-focused intervention in ASD in two folds. First, it filled the service gap for parents with older children emerging into adulthood. Despite a recognised dependency of adults with ASD on their parents, intervention empowering the parent role in this developmental stage was insufficient. Individuals with ASD in more advanced age are confronted with wanting more autonomy and continuously relying on a trusted person for the living arrangement. In such connection, interventions developed for parents of children with ASD may not be readily applicable to those with older children, as the interventions for the latter has to take a delicate balance between allowing autonomy and intervening. The CAPS solidified its age-appropriateness by embedding teaching contents in parent-child conflicts common to this age range. Further, the CAPS emphasises the importance of observing children with curiosity and patience in “to observe,” thus allowing room and space before parents decide to intervene.

Another clinical significance of the CAPS was its orientation to enhance PSE in parents of children with ASD through role competence. As discussed above, different psychological constructs may require different interventions. Interventions developed to enhance other psychological or behavioural outcomes may not guarantee a change in PSE and vice versa. The CAPS was a preliminary attempt to define and operationalise PSE and related parenting roles: to observe, reinforce, empathise and accompany, in the ASD population from our clinical experiences and research in ASD. It demonstrated that enhancing role competence through a two-pronged approach targeting both acquisition of parenting skills and cultivating positive attributes would be a possible intervention in enhancing PSE. The CAPS also serves as a prototype in blending essence of different treatment modalities, including positive behavioural support, mindfulness, developmental psychology and family life cycle, tailoring intervention for a specific population. With its demonstrated effectiveness, future research on the CAPS could examine the mediating effect of role competence on PSE with a larger sample size. Its applicability on parents with children of different ages or IQ levels under the autism spectrum could also be further explored.

### Limitations

Despite the novelty of the current study, it was limited by the use of a convenience sample of parents in a community centre. The validity of findings was potentially limited by a few factors in the methodology, including a lack of a control group, a lack of validated assessment on the four parent roles and a lack of long-term follow-up data. Further studies may extend the scope of research by adopting a randomised controlled design, a mediation analysis illustrating how the change in role competence contributes to PSE’s change and a more comprehensive assessment on potential constructs that mediate the change in PSE. The effectiveness of the CAPS was only examined among parents with children having an FSIQ of 70 or above. Its generalizability to the entire autism spectrum has yet to be confirmed. Modification and application to parents of children in young developmental stage or with other neurodevelopmental comorbidities, e.g., ADHD or intellectual disabilities, may be conducted to examine the intervention’s applicability across different ages, needs and symptom severity.

## Conclusion

The CAPS was a preliminary attempt to develop a parent intervention in the ASD population that aims to enhance parenting self-efficacy. The intervention filled the service gap for parents of adults with ASD. It addresses the role competence of an effective parent by employing a two-pronged training approach comprising cultivating positive attributes and acquisition of parenting skills. Drawing insights from positive behavioural support, mindfulness, developmental psychology and family life cycle, this innovative intervention may be further developed to serve different ages, needs and symptom severity in the ASD population.

## Data Availability Statement

The datasets presented in this article are not readily available because participants of the study were assured raw data would remain confidential and be stored in the hosting organization only. The data would not be shared. Requests to access the datasets should be directed to CL, leungnwcecilia@gmail.com.

## Ethics Statement

The studies involving human participants were reviewed and approved by New Life Psychiatric Rehabilitation Association, Hong Kong. The patients/participants provided their written informed consent to participate in this study.

## Author Contributions

RC conceived of the study. CL, BT, and DH participated in its design and coordination, developed the intervention protocol under the supervision of RC. CL performed data analysis and drafted the manuscript. All authors finalized the manuscript.

## Conflict of Interest

The authors declare that the research was conducted in the absence of any commercial or financial relationships that could be construed as a potential conflict of interest.

## Publisher’s Note

All claims expressed in this article are solely those of the authors and do not necessarily represent those of their affiliated organizations, or those of the publisher, the editors and the reviewers. Any product that may be evaluated in this article, or claim that may be made by its manufacturer, is not guaranteed or endorsed by the publisher.
